# Molecular Characterization of Avian Rotaviruses F and G Detected in Brazilian Poultry Flocks

**DOI:** 10.3390/v15051089

**Published:** 2023-04-29

**Authors:** Mariana S. Pinheiro, Juliana B. L. Dias, Melissa P. Petrucci, Carlos E. P. F. Travassos, Gabriella S. Mendes, Norma Santos

**Affiliations:** 1 Instituto de Microbiologia Paulo de Góes, Departamento de Virologia, Universidade Federal do Rio de Janeiro, Rio de Janeiro 21947-902, Brazil; pinheiromari10@gmail.com (M.S.P.); julianabldias@gmail.com (J.B.L.D.); gabriella_mendes@micro.ufrj.br (G.S.M.); 2 Centro de Ciências e Tecnologias Agropecuárias, Laboratório de Sanidade Animal, Universidade Estadual do Norte Fluminense Darcy Ribeiro, Campos dos Goytacazes 28013-602, Brazil; melpaesp@yahoo.com.br (M.P.P.); cacaetravassos@gmail.com (C.E.P.F.T.)

**Keywords:** rotavirus, RVF, RVG, chickens, genome sequencing

## Abstract

Avian rotaviruses (RVs) are important etiologic agents of gastroenteritis in birds. In general, avian RVs are understudied; consequently, there is a paucity of information regarding these viruses. Therefore, the characterization of these viral species is highly relevant because more robust information on genetic, epidemiologic, and evolutionary characteristics can clarify the importance of these infections, and inform efficient prevention and control measures. In this study, we describe partial genome characterizations of two avian RV species, RVF and RVG, detected in asymptomatic poultry flocks in Brazil. Complete or partial sequences of at least one of the genomic segments encoding VP1, VP2, VP4, VP6, VP7, NSP1, NSP4, NSP4, or NSP5 of 23 RVF and 3 RVG strains were obtained, and demonstrated that multiple variants of both RVF and RVG circulate among Brazilian poultry. In this study, new and important information regarding the genomic characteristics of RVF and RVG is described. In addition, the circulation of these viruses in the study region and the genetic variability of the strains detected are demonstrated. Thus, the data generated in this work should help in understanding the genetics and ecology of these viruses. Nonetheless, the availability of a greater number of sequences is necessary to advance the understanding of the evolution and zoonotic potential of these viruses.

## 1. Introduction

Rotaviruses (RVs) are members of the *Rotavirus* genus in the *Reoviridae* family and are divided into 12 species *A*–*L* [1,2,3,4,5]. Species *A*, *B*, *C*, and *H* (RVA, RVB, RVC, and RVH) infect both humans and animals; species *E* (RVE) has only been detected in pigs, while the other species have only been detected in other animal hosts. In addition, species *D*, *F*, and *G* (RVD, RVF, and RVG) have been detected in birds; species *I* (RVI) has been detected in canines and felines; species *J* (RVJ) in bats; and species *L* (RVL) in shrews [2,3,4,5].

The RV genome is composed of 11 segments of double-stranded RNA (dsRNA) encoding six structural proteins, VP1–VP4, VP6 and VP7, and five or six nonstructural proteins, NSP1–NSP5 and NSP6, depending on the viral strain [6,7]. The virion lacks a lipid envelope and measures approximately 75 nm in diameter. The genome is surrounded by three layers of proteins. The innermost layer consists of VP2, which surrounds the genome, and two other structural proteins, VP1 (RNA-dependent RNA polymerase, RdRp) and VP3 (responsible for capping RV ss(+)RNAs acting as mRNAs), comprising the core of the particle. VP6 forms the intermediate layer (intermediate capsid), which surrounds the core, thereby assembling into a double-layer particle (DLP). VP6 is the main structural component of the virion, and plays a fundamental role in the structure of the particle due to its interactions with the proteins of both the inner (VP2) and outer layers (outer capsid). The outer capsid that surrounds the DLP consists of VP7 and VP4. This last layer completes the virion, forming triple-layer particles [7]. Capsid spikes are formed by VP4, a cell-binding protein that facilitates viral internalization. This spike is inserted into the outer layer interacting with VP7. The globular domain of VP4 interacts with VP6 on the inner capsid surface. Interactions between VP4–VP7 and VP4–VP6 suggest that VP4 maintains the geometry of the inner and outer capsid, and also affects functional domains. VP7 interacts with VP6 to maintain viral structure [7]. Nonstructural proteins act at multiple stages of viral biosynthesis and interact with host proteins, influencing pathogenesis and immune response. NSP1 is an important regulator of innate immunity that inhibits the expression of type I interferon [8] and neutralizes nuclear factor kappa B [9]; NSP2 and NSP5 mediate viroplasm assembly [10,11]; NSP3 regulates the translation of RV mRNAs, linking them to the host cell machinery [12]; and NSP4, the RV enterotoxin, is essential for both pathogenesis and morphogenesis [13,14,15].

Avian RV infections were first described in 1977 in the United States, when virions resembling those of mammalian strains were visualized by electronic microscopy in the intestinal contents of turkeys with enteritis [16]. Subsequently, RV was also described in turkeys in the UK [17]. RVs have been isolated from other avian species, including chickens, pigeons, pheasants, parrots, gulls, partridges, and ducks [18]. Epidemiologic studies have shown that birds are frequently infected with RV, specifically by RVA, RVD, RVF, and RVG [14]. RVA and RVD have been detected frequently in sick birds of several species, while RVF and RVG have only been reported occasionally [19,20,21,22,23,24,25,26].

The evolution of RVs has been elucidated by analysis of the genetic repertoire of circulating strains, primarily by using classical Sanger sequencing. Consequently, several mechanisms, alone or in combination, that have been identified lead to RV evolution and diversity. Point mutations are among the main sources of diversity among these viruses [27,28], and the accumulation of these mutations can generate distinct genetic lineages [29]. Furthermore, gene rearrangement, reassortment of genomic segments, and interspecies transmission of partial or whole genomes are important evolutionary mechanisms described for RVs [28,30].

As established by the Rotavirus Classification Working Group (RCWG), the classification of RVA is based on the analysis of the 11 genomic RNA segments. Cutoff values have been defined for identifying the genotype of each segment [31,32,33]. This classification allowed the observation of the different natural mechanisms of evolution and highlighted the need to understand the genomic variability of these viruses better. A similar classification system has been proposed for RVB, RVC, and RVH [34,35,36,37]. Expanding this classification to other RV species is desirable; however, the limited number of published sequences confounds the establishment of robust cutoff values, especially for RVF and RVG, for which very few complete sequences are available. Factors that accentuate the difficulty of obtaining sequences are, in some cases, low viral loads in stool samples and the lack of a well-established viral isolation system for species other than RVA.

RVF and RVG were originally detected in chickens [38] and later in turkeys [39,40], pigeons [22], partridges, pheasants [41], gulls [24], and orange-winged parrots [42], and to date, have only been detected in birds [6,7]. Although detections of RVF and RVG in bird feces have been described in several countries [18], little is known about their genetic variability. Therefore, in this study, we describe partial genome characterizations of RVF and RVG strains detected in poultry in Brazil.

## 2. Materials and Methods

### 2.1. Samples

**Figure 1:**
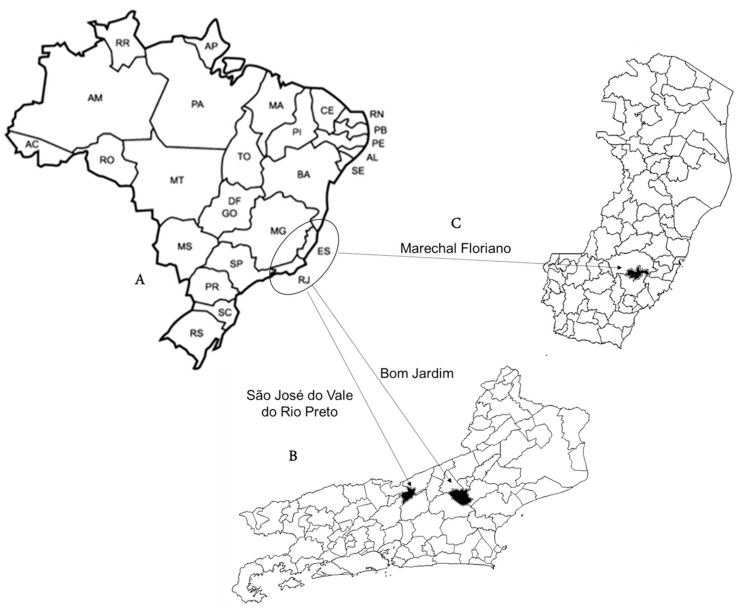
Map of Brazil showing the study region (circle; ES = Espírito Santo and RJ = Rio de Janeiro), located in the southeast of the country (**A**). Maps of the locations where fecal samples were obtained from birds analyzed in this study: (**B**) map of the state of Rio de Janeiro showing the cities of São José do Vale do Rio Preto in the East Region, and Bom Jardim in Bom Jardim in the Center Region of the state; (**C**) map of the state of Espírito Santo showing the city of Marechal Floriano in the Southwest Region of the state.

Fecal samples included in this study were from a collection housed at the Laboratory of Respiratory, Enteric and Ocular Viruses (LAVIREO) at the Federal University of Rio de Janeiro. Samples in this collection were obtained from asymptomatic chickens in the southeastern Brazilian cities of São José do Vale do Rio Preto (May 2013), and Bom Jardim (October 2018), in the state of Rio de Janeiro, and Marechal Floriano (October 2018), in the state of Espírito Santo (Figure 1), and were analyzed previously for RVF and RVG by RT-PCR [43].

### 2.2. RNA Extraction, Amplification, and Sequencing

Stool suspensions were prepared in 10% (*w*/*v*) phosphate-buffered saline (pH 7.2) and then centrifuged at 2500× *g* for 5 min; viral dsRNA was extracted from 300 μL of the supernatant using the guanidine isothiocyanate-phenol-chloroform method [44]. RV cDNA was synthesized and amplified using specific primers (Appendix A Table A1) designed according to RVF and RVG sequences available in GenBank. The amplified genomic segments were sent to Macrogen Inc. (Seoul, Republic of Korea) for sequencing. Overlapping sequences were assembled and edited using SeqMan, EditSeq, and MegAlign in the Lasergene software package (DNASTAR, Madison, WI, USA). Phylogenetic analysis was performed with MEGA software (version 11.0.11) [45] and aligned with corresponding sequences obtained from GenBank (https://www.ncbi.nlm.nih.gov/nucleotide/accessed on 21 March 2023). Access codes of the reference strains are presented in Appendix A Table A2. Dendrograms were constructed using the maximum likelihood method and the Kimura 2-parameter model. Statistical significance was estimated by bootstrap analysis with 1000 pseudoreplicates. Sequences generated in this study were deposited in GenBank under accession numbers OL688642, OL688643, OL688646, OL688659-OL688661, and OQ627756–OQ627800.

## 3. Results

Complete or partial sequences of at least one of the genomic segments encoding VP1, VP2, VP4, VP6, VP7, NSP1, NSP2, NSP4, or NSP5 of 23 RVF and 3 RVG strains were obtained (Appendix A Table A3).

### 3.1. Analysis of RVF Strains

#### 3.1.1. Genomic Segment Encoding VP1

**Figure 2:**
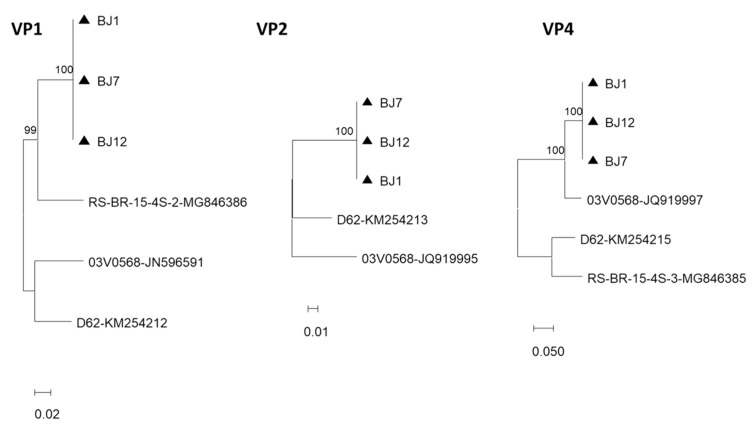

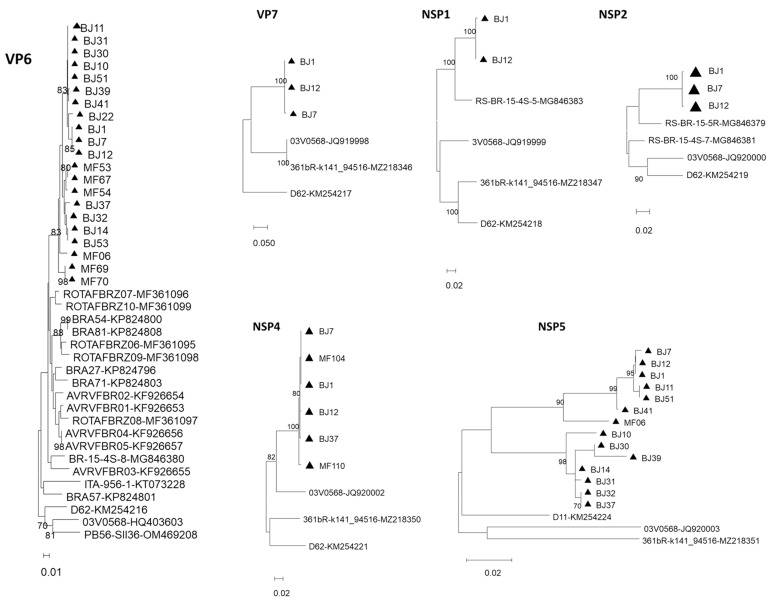
Dendrograms constructed from complete or partial sequences of the VP1, VP2, VP4, VP6, VP7, NSP1, NSP2, NSP4, and NSP5-encoding genome segments of RVF strains. Distances were corrected using the Kimura 2-parameter model. The dendrogram was constructed using the maximum likelihood method. Statistical support was provided by bootstrapping 1000 pseudoreplicates. Bootstrap values above 70% are given as branch nodes. GenBank reference strain accession numbers are shown next to the strain identification. RVF strains detected in this study are indicated by black triangles.

Complete or partial sequences of the open reading frame (ORF) of gene segment 1, encoding VP1, were obtained from three strains detected in Bom Jardim, Rio de Janeiro. A comparison of the complete sequence of the BJ12 strain, and reference strains from Germany and the Republic of Korea showed nucleotide identities of 88.7% and 89.7%, respectively, and amino acid identities of 96% and 97%, respectively. Partial sequences were obtained for strains BJ1 and BJ7 from Bom Jardim. To facilitate the analysis, all sequences were edited to the length of 1115 bp, which permitted the analysis of the three strains from Rio de Janeiro and reference strains from Germany and South Korea, and other RVF strains detected previously in Brazil (Figure 2). The comparison of the partial sequences of the three strains detected in Bom Jardim showed 100% identity and that they were closer to the Brazilian RS-15-4S-2 strain (90.5% identity), detected in the state of Rio Grande do Sul, southern Brazil, than to the strains from Germany (03V0568; 88.4%) and Republic of Korea (D62; 89.7%).

#### 3.1.2. Genomic Segment Encoding VP2

Partial sequences of gene segment 2, encoding VP2 of three strains, BJ1, BJ7, and BJ12, were obtained. For phylogenetic analysis, the sequences were edited to the length of 1200 bp (Figure 2). The strains detected in this study had 99.4 to 100% identity among themselves, and 88.7% to 90.7% nucleotide identity with German and Republic of Korean reference strains, respectively.

#### 3.1.3. Genomic Segment Encoding VP4

Complete sequences of the ORFs of gene segment 3, which encodes VP4, of two strains, BJ1 and BJ7, were obtained. Phylogenetic analysis showed high nucleotide (99.9%) and amino acid (100%) identities. Comparison with reference strains showed that the strains from Bom Jardim were closer to the German strain 03V0568, with percentage identities of nucleotides and amino acids of 96.1% and 96.8%, respectively. Identities with the Republic of Korean strain (D62) were 78.1% for nucleotides and 83.7% for amino acids. Interestingly, another Brazilian strain from Rio Grande do Sul, RS-BR-14-4S-3, was closer to the German and Republic of Korea strains than to the strains from Bom Jardim.

The partial sequences of BJ1, BJ7, and BJ12 strains were analyzed by adjusting all sequence lengths to 1130 bp (Figure 2). Phylogenetic analyses showed that the strains detected in this study had 99.9 to 100% identity with each other, and were more closely related to the reference strains from Germany than to those from South Korea and Brazil.

#### 3.1.4. Genomic Segment Encoding VP6

Complete sequences of the OFR of gene segment 6, encoding VP6, were obtained for the BJ1, BJ7, and BJ12 strains. Phylogenetic analysis showed that the strains detected in this study were identical. Comparison with German (03V0568) and Swiss (PB56-SII36) reference strains showed nucleotide identities of 88.1–85.8% and amino acid identities of 95.7–92.1%, respectively, with RVF from Bom Jardim. On the other hand, the comparison with a Brazilian strain detected in the state of Rio Grande do Sul showed 91.9% nucleotide and 99.7% amino acid identities.

In addition, we obtained the partial VP6 sequences of 18 RVF strains from both Bom Jardim in the state of Rio de Janeiro and Marechal Floriano in the state of Espírito Santo. Sequences were adjusted to the size of 280 bp (Figure 2). Phylogenetic analysis showed that our strains presented greater genetic proximity to RVFs previously detected in Brazil, although they form a separate clade than those detected in Germany (03V0568), the Republic of Korea (D62), and Switzerland (PB56-SI36). Interestingly, the BRA57 strain, detected in the state of Pará, northern Brazil, grouped separately with a partridge strain (956_1) detected in Italy.

#### 3.1.5. Genomic Segment Encoding VP7

Partial sequences of gene segment 9, which encodes VP7, were obtained for strains BJ1, BJ7, and BJ12. Sequences were adjusted to 398 bp, which covered the same ORF regions of all samples (Figure 2). Phylogenetic analysis showed that the strains detected in this study had 99.2 to 100% identity. Comparison with German (03V0568), Chinese (361BR-K141_94516), and Republic of Korean (D62) strains showed identities of 75.9% to 80.4%.

#### 3.1.6. Genomic Segment Encoding NSP1

The complete sequence of segment 5, which encodes NSP1, was obtained for BJ12. A partial sequence was determined for BJ1. Sequence lengths were adjusted to 997 bp to include both strains (Figure 2). Phylogenetic analyzes showed that the BJ strains presented 99.7% nucleotide identity and were closer to the Brazilian strain from Rio Grande do Sul, BR-15-4S-5 (91.5–91.9%) than RVF strains from China (361BR-K141_94516), Germany (03V0568), and South Korea (D62) (85.6–86.9%).

#### 3.1.7. Genomic Segment Encoding NSP2

Sequence of the complete ORF of gene segment 8, encoding the NSP2 of the BJ1 strain, and partial sequences of the BJ7 and BJ12 strains were obtained. The sequence analysis of the BJ1 ORF disclosed an identity of 88.6% and 92.8% of nucleotides and amino acids, respectively, with the German reference strain (03V0568), and 90.3% and 95% with two Brazilian strains (RS-BR-15-4S-7 and RS-BR-15-5R) detected in Rio Grande do Sul. When the length of the sequences was adjusted to 594 bp (Figure 2), phylogenetic analysis showed that they grouped in a single clade close to, but distinct from, other strains detected in southern Brazil. This could suggest the independent evolution of the RVF strains circulating in the country. However, the analysis of the complete ORFs of a larger number of samples is necessary to confirm this hypothesis.

#### 3.1.8. Genomic Segment Encoding NSP4

Complete sequences of the ORF of gene segment 11, which encodes NSP4, were obtained for six strains, BJ1, BJ7, BJ12, and BJ37 from Rio de Janeiro, and MF104 and MF110 from Espírito Santo (Figure 2). Phylogenetic analyses showed that the strains detected in this study presented high nucleotide and amino acid identity. Comparison with reference strains from Germany (03V0568), China (361bR-k141_94516), and the Republic of Korea (D62) showed that the Brazilian strains had nucleotide identities of 88.4–88.6% and amino acid identities of 88.9–91.8%. Unfortunately, genetic information regarding NSP4 of other Brazilian RVF strains is not available.

#### 3.1.9. Genomic Segment Encoding NSP5

The complete ORF of gene segment 10, encoding NSP5, was obtained for five RVF strains: BJ1, BJ7, BJ10, BJ11, and BJ12. Phylogenetic analyses showed that the BJ strains had 91.2–100% nucleotide identity and 94.5–100% amino acid identity among themselves. Comparison with reference strains from Germany, China, and the Republic of Korea showed that the strains from Rio de Janeiro showed nucleotide identities of 86.7% to 89.5%, respectively, and amino acid identities of 89.8% and 94.5%, respectively. Adjusting the length of the sequences to 400 bp enabled the analysis of eight more strains: BJ14, BJ30, BJ31, BJ32, BJ37, BJ39, BJ41, and BJ51 detected in Bom Jardim, and MF06 detected in Marechal Floriano. The dendrogram shows that the Brazilian strains were closely related but not identical (Figure 2).

## 4. Analysis of RVG Strains

### 4.1. Genomic Segment Encoding VP6

**Figure 3:**
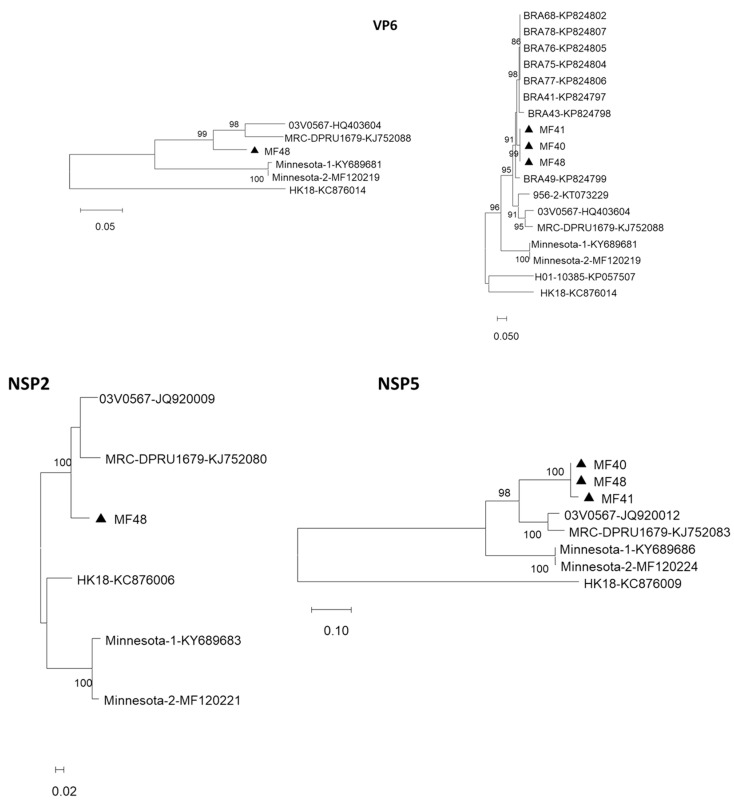
Dendrograms constructed from complete or partial sequences of the VP6, NSP2, and NSP5-encoding genome segments of RVG strains. Distances were corrected using the Kimura 2-parameter model, and the dendrogram was constructed using the maximum likelihood method. Statistical support was provided by bootstrapping 1000 pseudoreplicates. Bootstrap values above 70% are given as branch nodes. GenBank reference strain accession numbers are shown next to the strain identification. RVG strains detected in this study are indicated by black triangles.

Complete or partial sequences of the sixth segment encoding VP6 of three RVG strains detected in Marechal Floriano were obtained: MF48 (complete ORF), MF40, and MF41 (partial ORFs). When comparing the complete ORF sequence of the MF48 strain with the reference strains that are available on GenBank, we observed that the strain from Brazil is genetically closer to chicken strains from South Africa (MRC-DPRU1679) and Germany (03V0568), with 89% and 89.3% nucleotide identity, and 96.9 and 97.7% amino acid identity, respectively. It has 80% and 89% nucleotide and amino acid identity, respectively, with the US turkey strains (Minnesota-1 and Minnesota-2) and is phylogenetically distant from the Hong Kong pigeon strain (HK18) (Figure 3). When we adjusted the sequence length to 642 bp, we were able to include the MF40 and MF41 strains in the analysis (Figure 3). The dendrogram showed that the strains detected in Brazil were genetically similar (94.5 to 96.9% identity) yet grouped differently to form three sub-clades: the strains from Espírito Santo (MF40, MF41 and MF48), detected in 2018, grouped in one clade and the strains from Rio Grande do Sul, detected in 2009, grouped in two different clades. These data suggest that the Brazilian strains have a common ancestor and possibly evolved independently of each other, leading to the observation of different variants circulating in our country. Partial sequences from a partridge strain (956-2) from Italy and a gull strain (H01-10385) from the Netherlands were also included in this analysis. The phylogenetic analysis showed a separation between strains of different host species, except for the partridge strain that grouped with chicken samples. These findings suggest (i) that different VP6 genotypes circulate among RVG strains and (ii) that these genotypes may be associated with different hosts (chickens, turkeys, and pigeons).

### 4.2. Genomic Segment Encoding NSP2

A partial sequence of the eighth gene segment that encodes NSP2 was obtained for the MF48 RVG strain (Figure 2) and showed 89.6–90.1% identity with the RVG strains detected in chickens, 84.7% with the pigeon strain, and 80.4% with turkey strains.

### 4.3. Genomic Segment Encoding NSP5

Three complete ORFs of gene segment 11, encoding the NSP5, from strains MF40, MF41, and MF48 were obtained. Phylogenetic analysis showed that, similar to the findings of VP6, the Brazilian strains were genetically closer to chicken strains and once again separated into different clades according to host species (Figure 2).

## 5. Discussion

RVs are the most prevalent etiologic agents of diarrhea among diverse hosts that include mammals and birds [6]. Avian RVs have great epizootic potential and can thereby bring economic losses to poultry farming. Despite their importance, they are poorly studied, and consequently, genomic and epidemiologic information regarding these viruses, especially RVF and RVG, is limited. Genomic characterization of circulating strains is necessary for accurate identification and classification, and to provide a better understanding of viral evolution [30,46,47,48]. Moreover, knowledge of genetic diversity is important to inform the prevention and control of viral infections. For these reasons, genomic sequencing studies have been conducted to demonstrate the wide diversity of RVs. However, most data in the literature on RV are related to RVA, underscoring the need to study other species.

In this study, we obtained the complete ORF sequences of the genome segments that encode VP6, NSP2, NSP4, and NSP5 of RVF strains circulating in two southeastern states of Brazil: Rio de Janeiro and Espírito Santos. When compared to the sequences of reference strains, they presented nucleotide identity values for VP6, NSP2, and NSP4 greater than 85%, the suggested cutoff value for RVA. The application of the same criteria to RVF would suggest that these belong to the same genotype. However, it is important to point out that the Brazilian strains grouped in a distinct clade in the dendrograms, suggesting their independent evolution.

On the other hand, the percentages of nucleotide identity observed for NSP5 could suggest the circulation of different genotypes, as they are below 91%, which is the cutoff value for this gene in the case of RVA. Furthermore, analysis of partial NSP5 sequences showed that although the strains in this study clustered closely, they are not all in the same clade. This difference may have arisen through the typical mechanisms of RV evolution, primarily through point mutations. The MF06 strain originating from Espírito Santo presented similarity with the strains from Rio de Janeiro; however, its distribution in the dendrogram shows segregation in a specific clade, suggesting the circulation of separate variants in different Brazilian states.

When analyzing partial sequences, we observed that for VP1, using a fragment that corresponded to 54% of the ORF, the Brazilian strains and the reference strains shared >83% identity, a cutoff value stipulated to differentiate the VP1 genotypes from RVA. For genes encoding VP2, VP4, VP7, and NSP1, the sequences corresponded to <50% of the ORF (20%, 20%, 45%, and 40%, respectively). In the case of genes encoding VP2 and NSP1, the identity between all strains was well above a cutoff value of 84%, suggesting a single genotype. However, for VP4, the nucleotide identity values and the topology of the dendrogram suggest two distinct genotypes: one represented by the strain from Germany and the strains from Rio de Janeiro, and a second represented by the strain from South Korea and the strain from Rio Grande do Sul. Interestingly, these data suggest a distinct origin of the strains from Rio de Janeiro and Rio Grande do Sul. In the case of VP7, the results suggest three different genotypes represented by the Brazilian strains, the strains from Germany and China, and the strain from South Korea.

VP4 and VP7 are responsible for cell entry and the induction of neutralizing antibodies and, therefore, represent the main targets for vaccine development [49]. Consequently, an understanding of the genetic characteristics of these proteins is important. Unfortunately, sequences of VP7 from Brazilian strains have not been obtained, and only a partial sequence of VP4 is available. The possibility of varying genotypes of VP4 and VP7 carries extremely important implications for vaccine development, as changes in the antigenicity of these proteins can impact the generation of neutralizing antibodies. No vaccines are available to prevent avian rotavirus disease; consequently, an understanding of the different circulating variants is essential to the future development of an effective vaccine. At present, the need to develop specific vaccines against infections caused by RVF and RVG has not yet been established. Although this virus has been detected in different species of birds, with and without disease symptoms, knowledge of its epidemiology and pathogenic importance is still incipient to allow such an evaluation. On the other hand, RVA and RVD species are already established as important disease agents in birds [18].

Overall, phylogenetic analyzes of all sequenced RVF genome segments demonstrated that Brazilian strains, although possibly having a common ancestor, evolved independently, leading to different variants circulating in the country.

As for RVG strains, three genes, VP6, NSP2, and NSP5, were sequenced. Analysis of the complete and partial ORFs of VP6 suggests the existence of different genotypes and possibly the segregation of these genotypes according to host species. Furthermore, the circulation of different variants in Rio de Janeiro and Rio Grande do Sul was also demonstrated. This evidence was reinforced by the analysis of NSP2 and NSP5.

The RCWG proposal for RVA defines the cutoff values for the genetic classification of individual genes. However, for the application of these criteria, the RCWG imposes conditions for the identification of genotypes. Therefore, no genotype should be assigned based on less than 500 nucleotides or less than 50% of the ORF sequence. As the percentage cutoff values for each of the 11 genomic segments were calculated based on complete ORFs, applying these cutoff percentages to a partial ORF sequence can lead to erroneous conclusions. Only under certain circumstances, when all three of the following restrictions are met, can a partial gene sequence be used to assign an already established genotype to a given RVA gene: (1) at least 50% of the ORF gene sequence must be determined; (2) at least 500 nucleotides of ORF must be determined; and (3) the identity between the analyzed strain and the reference strains belonging to an established genotype must be at least 2% above the cutoff value. New genotypes can be identified only by analyzing the complete ORF of the gene in question [31]. The extremely small number of available RVF and RVG genomic sequences, particularly complete ORF sequences, impedes the establishment of reliable cutoff values for the determination of genotypes of these species. Thus, our analyses have theoretical value, intended only to demonstrate variants within these viral species, which, in the future, may or may not be defined as different genotypes.

Birds are recognized as important reservoirs of viruses that can threaten human health, such as the influenza A virus, which infects both birds and mammals, enabling the reassortment of genomic segments and changes in tropism and transmission efficiency [50]. Influenza viruses and RVs are both zoonotic pathogens with segmented RNA genomes, suggesting that the same phenomena could occur in RVs. The zoonotic potential of RVs that infect mammals, particularly RVA and RVC, has already been demonstrated unequivocally [30,51]. On the other hand, the zoonotic potential of avian RVs has yet to be explored. Thus, further studies will be essential to understand better the molecular characteristics and evolution of RV species that infect birds.

In this study, new and important information regarding the genomic characteristics of RVF and RVG was described, despite the small number of characterized strains. These difficulties can be partially explained by the samples used for amplification of the viral genomes, which came from asymptomatic chickens and therefore contained low viral loads, as well as the limited number of available RVF and RVG sequences, which confounded the selection of more efficient primers. However, despite the limitations of this study, the circulation of RVF and RVG variants among poultry was clearly demonstrated. Nonetheless, the availability of a greater number of sequences is necessary for a better understanding of the evolution and zoonotic potential of these viruses.

## Data Availability

The nucleotide sequences of the 2014–2018 viruses obtained in this study were submitted to the GenBank database under accession numbers OL688642, OL688643, OL688646, OL688659-OL688661, and OQ627756-OQ627800.

## Appendix A

**Table A1:** Primers used for sequencing the RVF and RVG strains.

Primer	Primer Sequence 5′ → 3′	Gene	Position ^†^
RFVP1.F1	ATTAATTGCGTACGATGGGGA	VP1	5–25
RFVP1.F2	GGATCCTGCAATATCTACATC		429–449
RFVP1.F3	GCAAATGTWTATTCATGGTC		991–1010
RFVP1.F4	TGCTTAAAGATGAYGTTATTA		1145–1165
RFVP1.F5	GGTAGAAGRGATGTACCAGGT		1372–1392
RFVP1.R1	TTAATYATTTTTGATGAATA		835–854
RFVP1.R2	ACTTCCAAATTTTATTTCC		1242–1260
RFVP1.R3	GCTAAATTTGCAATAGAATTA		1807–1827
RFVP1.R4	TAACRTTCATAAGTGCATAAG		1985–2005
RFVP1.R5	CCTGATATYTTAACTGATGTC		2229–2249
RFVP1.R6	GATARTAAATCATAAACATATG		2984–3005
RFVP1.R7	ATTATTGCAGCTTACTCTTGG		3270–3290
RFVP2.F1	GGCTATTATTGGCAGGATGT	VP2	1–20
RFVP2.F2	GCAGARATGAGRCATAGAGT		764–783
RFVP2.F3	GGGAAACATTAACGTTAACT		1041–1060
RFVP2.F4	GTAATGAATCAGCAATATGC		1772–1791
RFVP2.R1	CAGATTATAAGGAACATATC		978–997
RFVP2.R2	CTAACTATATTAGCTTCAGC		788–807
RFVP2.R3	AGAGCTAATACAAACATTCC		1307–1326
RFVP2.R4	GATGCATCAGGATATACAGC		1862–1881
RFVP2.R5	GGAGATAATACTCCTCCAGC		2741–2760
RFVP4.F1	GATGGCTTCTCGCTTTTGGG	VP4	11–30
RFVP4.F2	AAATGCGGTAGTTTTGGAATAG		352–372
RFVP4.F3	GGTTATCAATTTTCAACGTCT		787–809
RFVP4.F4	GTAGGAAGTATAACTCCATA		1104–1123
RFVP4.F5	ATCAAGRTGGCAGAAAARTT		1500–1519
RFVP4.R1	CATATACCATGTTTCTGAAT		1010–1029
RFVP4.R2	AATCCAGCTTGTGACACGAC		690–709
RFVP4.R3	AGGATAATATGGAGTTATACT		1110–1130
RFVP4.R4	TTGAAT TCCTGTCGTAATTGG		1480–1500
RFVP4.R5	TATGCTATATTAGCATCCTTA		1980–2000
RFVP4.R6	ATACTGTTTCTCGCTCAAAGT		2250–2270
RFVP6.F1	GGCTTATAAAAGTCAATCAG	VP6	1–20
RFVP6.F2	AAGTCAATCAGTCGCAATGG		10–29
RFVP6.F3	CGTACAGAACCAGGTCAAATGTG		620–642
RFVP6.F4	GCCAGTGCCAATATCTGATGC		667–687
F5.RVF	ATGTGAAACTGAAATGTGTCTTGAATC		310–336
F6.RVF	TACAAAAAGTTAGAGTTAGAACAGCTTA		447–474
RFVP6.F7	GGTCTACTTAATGATCAARTAC		730–737
RFVP6.F8	CAATACAAGTTGATACTGATGC		690–710
RFVP6.R1	CCTGCYACATCATCCATAGC		539–558
R2.RVF	TTGATCATTAAGTAGACCAGAYGCATC		707–733
RFVP6.R3	AGCTGTTCTAACTCTAACTT		453–472
RFVP6.R4	CAGTGATACTATCGGAATAAACCA		1241–1264
RFVP6.R5	CACAGTTGCCCGGCCAAACG		1268–1287
RFVP6.R6	GCATATTATCTTGTCTAGAT		1150–1169
RFVP7.F1	CGAACAGCCTCCATCAGCTCGTGT	VP7	17–40
RFVP7.F2	CCTGTAATTCAGGATGTTTGCTG		57–79
RFVP7.F2	GTARCCTGTAATTCAGGATGTT		53–74
RFVP7.F3	GAAGTAAATGAGTTATTTCAAT		355–376
RFVP7.F4	TCGACTGATGTGAAAACATATG		601–622
RFVP7.F4	TCAACTGATGCTACTACATATG		601–622
RFVP7.F5	TATAGAATGAATATTACTGG		673–692
RFVP7.R1	GGTCATAATGTTGTTCGCAAC		970–990
RFVP7.R2	CAACGTTAATGATTATTTATC		953–973
RFVP7.R3	GAGGAATAATCTGGTCCAAC		733–752
RFVP7.R4	GATAAGTCATCTGGTCCATG		412–431
RFVP7.R4	GATAAATCACTCGGCCCAT		413–431
RFNSP1.F1	GTG TGC CGA TTC AGA GAT GG	NSP1	23–42
RFNSP1.F2	GGT ATG AGT GTA GTW CCA GC		711–730
RFNSP1.F3	CAA TTY CGT GAT TGG AA		1176–1192
RFNSP1.R1	CCG TGT GCG ATG CTG AAT CGG		1745–1765
RFNSP1.R2	GTA TAC TCA CAA TCT TAT CAT		792–812
RFNSP1.R3	ACT AAT CAR TCA CCT CTT AT		1401–1420
RFNSP2.F1	TTRTTTTTGATATAGAGCAGT	NSP2	10–30
RFNSP2.F3	GGTCCGGCAAAATCCTGCCTGCCG		28–51
RFNSP2.F4	GAATGATGCAGAAGATAGAC		412–431
RFNSP2.F5	GAGTATAAAATTACATTCAA		608–627
RFNSP2.R1	GGTCGTAGTATGATATAGAT		1049–1068
RFNSP2.R2	TTGCTTTAATAGCTTTRAGAG		786–806
RFNSP2.R3	GTACTTATTCATATATTCAATA		529–550
RFNSP2.R4	GTTTCAGGATTAAGTTATTGGG		1019–1040
RFNSP4.F1	CCTCATCTTAGTTATACGTAC	NSP4	11–31
RFNSP4.F2	CCCTCAGTGGTTTTGACAAG		31–50
RFNSP4.R1	GGTCATAACTCATCCGTTAG		659–678
RFNSP4.R2	GCTGATCGCTGCACTCTGG		641–659
RFNSP4.R3	GGTACAGTATGTTATACGC		607–621
RFNSP4.R4	ACCACTAGCATCTTCAGTTC		406–425
RFNSP5.F1	GAGCATGGATCTTGATATAGAC	NSP5	20–42
RFNSP5.F2	ATGGAATCTGTAAATAAT		303–320
RFNSP5.F3	TCAGTTAAGTCATCAAATTC		357–376
RFNSP5.R1	CATGATTATAGATCGGATATAAGC		656–679
RFNSP5.R2	GCTGAGTAATGCTTTGCACTGC		411–432
RGVP6.F1	GGAAAGAAATCTCCAACCTAG	VP6	1–21
RGVP6.F2	GCTAAGCTCGAACCTCAAATT		459–479
772F-RVG	CAGATATGGCGAGRGGTGAT		772–791
RGVP6.R1	AATTCTATTACTATATCACC		786–805
RGVP6.R2	AGCTTAGCTGTATCATATGC		447–466
1229R-RVG	AAACTCTCCTCCACAGCCGA		1229–1248
RGNSP2.F1	GAGTGCGTCGTGAGAAGGGAG	NSP2	20–40
RGNSP2.F3	GTTTTTGARGATGTATTTGAA		380–400
RGNSP2.R1	CAGCGCTCAATGAATGGATTT		978–998
RGNSP2.R3	GTACTGTTCTAACATGTCCGT		750–770
RGNSP4.F3	GATKCATTTAAGATTCTTT	NSP4	105–123
RGNSP4.F4	CARAATTCGAAGGAAATGGT		384–403
RGNPS4.R3	AACCCATCGGCTCTGCACC		740–758
RGNSP4.R4	TCCATYGATTCTTCAATTCC		534–553
RGNSP5.F1	GGAATATTAAAGTGTCGCTTGGTG	NSP5	1–24
RGNSP5.F2	GGTGGCTGGAAACACTGAGTGG		21–42
RGNSP5.R1	CCCAGAAATAATCAGTGGAGTC		657–678
RGNSP5.R2	TCAACTTCATCHGCCCAGTT		315–334
RGNSP5.R3	CTAATTTTCGAWATTTCAT		445–463

^†^ Based on sequences from strains RVF Ch-03V0568, RVG Ch-03V0567 and MRC-DPRU1679.

**Table A2:** RVF and RVG reference strains GenBank access code.

Viral Strain	Origin	Gene										
		VP1	VP2	VP3	VP4	VP6	VP7	NSP1	NSP2	NSP3	NSP4	NSP5
RVF												
Ch-03V0568	Germany	JN596591	JQ919995		JQ919997	HQ403603	JQ919998	JQ919999	JQ920000		JQ920002	JQ920003
Ch-361bR-k141_94516	China						MZ218346				MZ218350	MZ218351
Ch-D62	South Korea	KM254212	KM254213		KM254215	KM254216	KM254217	KM254218	KM254219		KM254221	KM254222
Ch-D11	South Korea											KM254224
Ch-PB56-SII36	Switzerland					OM469208						
Ch-ITA-956-1	Italy					KT073228						
Ch-BR-15-4S-8	Brazil					MG846380						
Ch-RS-BR-15-4S-7	Brazil								MG846381			
Ch-RS-BR-15-5R	Brazil								MG846379			
Ch-AVRVFBR01	Brazil					KF926653						
Ch-AVRVFBR02	Brazil					KF926654						
Ch-AVRVFBR03	Brazil					KF926655						
Ch-AVRVFBR04	Brazil					KF926656						
Ch-AVRVFBR05	Brazil					KF926657						
Ch-BRA27	Brazil					KP824796						
Ch-BRA54	Brazil					KP824800						
Ch-BRA57	Brazil					KP824801						
Ch-BRA71	Brazil					KP824803						
Ch-BRA81	Brazil					KP824808						
RVG												
Ch-03V0567	Germany					HQ403604						JQ920012
Ch-MRC-DPRU1679	South Africa					KJ752088						KJ752083
Ty-Minnesota-1	USA					KY689681						
Ty-Minnesota-2	USA					MF120219						
Pg-HK18	Hong Kong					KC876014						
Hg-H01-10385	Netherlands					KP057507						
Pr-956-2	Italy					KT073229						
Ch-BRA41	Brazil					KP824797						
Ch-BRA43	Brazil					KP824798						
Ch-BRA49	Brazil					KP824799						
Ch-BRA68	Brazil					KP824802						
Ch-BRA75	Brazil					KP824804						
Ch-BRA76	Brazil					KP824805						
Ch-BRA77	Brazil					KP824806						
Ch-BRA78	Brazil					KP824807						

Ch = chicken; Ty = turkey; Pg = pigeon; Hg = herring gull; Pr = partridge.

**Table A3:** Length of fragments analyzed for each RVF and RVG gene.

Gene Segment	Virus Strains	
	RVF	RVG
VP1	Complete ORF (3258 bp): BJ12 Partial ORF (1115 bp): BJ1, BJ7	-
VP2	Partial ORF (1200 bp): BJ1, BJ7, BJ12	-
VP3	-	-
VP4	Complete ORF (2204 bp): BJ1, BJ7 Partial ORF (1130 bp): BJ12	-
VP6	Complete ORF (1188 bp): BJ1, BJ7, BJ12 Partial ORF (280 bp): BJ10, BJ11, BJ14, BJ22, BJ30, BJ31, BJ32, BJ37, BJ39, BJ41, BJ51, BJ53, MF06, MF53, MF54, MF67, MF69, MF70	Complete ORF: MF48 (1173 bp) Partial ORF (642 bp): MF40, MF41
VP7	Partial ORF (398 bp): BJ1, BJ7, BJ12	-
NSP1	Complete ORF (1641 bp): BJ12 Partial ORF (997 bp): BJ1	-
NSP2	Complete ORF (954 bp): BJ1 Partial ORF (594 bp): BJ7, BJ12	Partial ORF (953 bp): MF48
NSP3	-	-
NSP4	Complete ORF (507 bp): BJ1, BJ7, BJ12, BJ37, MF104, MF110	-
NSP5	Complete ORF (654 bp): BJ1, BJ7, BJ10, BJ11, BJ12 Partial ORF (400 bp): BJ14, BJ30, BJ31, BJ32, BJ37, BJ39, BJ41, BJ51, MFO6	Complete ORF (543 bp): MF40, MF41, MF48

## References

[1] Matthijnssens J., Otto P.H., Ciarlet M., Desselberger U., Van Ranst M., Johne R. (2012). VP6-sequence-based cutoff values as a criterion for rotavirus species demarcation. Arch. Virol..

[2] Mihalov-Kovács E., Gellért Á., Marton S., Farkas S.L., Fehér E., Oldal M., Jakab F., Martella V., Bányai K. (2015). Candidate new rotavirus species in sheltered dogs, Hungary. Emerg. Infect. Dis..

[3] Bányai K., Kemenesi G., Budinski I., Földes F., Zana B., Marton S., Varga-Kugler R., Oldal M., Kurucz K., Jakab F. (2017). Candidate new rotavirus species in Schreiber’s bats, Serbia. Infect. Genet. Evol..

[4] Johne R., Tausch S.H., Grützke J., Falkenhagen A., Patzina-Mehling C., Beer M., Höper D., Ulrich R.G. (2019). Distantly Related Rotaviruses in Common Shrews, Germany, 2004–2014. Emerg. Infect. Dis..

[5] Johne R., Schilling-Loeffler K., Ulrich R.G., Tausch S.H. (2022). Whole Genome Sequence Analysis of a Prototype Strain of the Novel Putative Rotavirus Species L. Viruses.

[6] Estes M.K., Greenberg H.B., Knipe D.M., Howley P.M., Cohen J.I., Griffin D.E., Lamb R.A., Martin M.A., Racaniello V.R., Rizman B. (2013). Rotaviruses. Fields Virology.

[7] Desselberger U. (2014). Rotaviruses. Virus Res..

[8] Barro M., Patton J.T. (2007). Rotavirus NSP1 inhibits expression of type I interferon by antagonizing the function of interferon regulatory factors IRF3, IRF5, and IRF7. J. Virol..

[9] Graff J.W., Ettayebi K., Hardy M.E. (2009). Rotavirus NSP1 inhibits NFkappaB activation by inducing proteasome-dependent degradation of beta-TrCP: A novel mechanism of IFN antagonism. PLoS Pathog..

[10] Criglar J.M., Hu L., Crawford S.E., Hyser J.M., Broughman J.R., Prasad B.V., Estes M.K. (2014). A novel form of rotavirus NSP2 and phosphorylation-dependent NSP2-NSP5 interactions are associated with viroplasm assembly. J. Virol..

[11] Geiger F., Acker J., Papa G., Wang X., Arter W.E., Saar K.L., Erkamp N.A., Qi R., Bravo J.P., Strauss S. (2021). Liquid-liquid phase separation underpins the formation of replication factories in rotaviruses. EMBO J..

[12] Vende P., Piron M., Castagné N., Poncet D. (2000). Efficient translation of rotavirus mRNA requires simultaneous interaction of NSP3 with the eukaryotic translation initiation factor eIF4G and the mRNA 3’ end. J. Virol..

[13] Ball J.M., Mitchell D.M., Gibbons T.F., Parr R.D. (2005). Rotavirus NSP4: A multifunctional viral enterotoxin. Viral Immunol..

[14] Hyser J.M., Collinson-Pautz M.R., Utama B., Estes M.K. (2010). Rotavirus disrupts calcium homeostasis by NSP4 viroporin activity. mBio.

[15] Chang-Graham A.L., Perry J.L., Strtak A.C., Ramachandran N.K., Criglar J.M., Philip A.A., Patton J.T., Estes M.K., Hyser J.M. (2019). Rotavirus Calcium Dysregulation Manifests as Dynamic Calcium Signaling in the Cytoplasm and Endoplasmic Reticulum. Sci. Rep..

[16] Bergeland M.E., McAdaragh J.P., Stotz I. (1977). Rotaviral enteritis in turkey poults. Proceedings of the 26th Western Poultry Diseases Conference.

[17] McNulty M.S., Allan G.M., Stuart J.C. (1978). Rotavirus infection in avian species. Vet. Rec..

[18] Dhama K., Saminathan M., Karthik K., Tiwari R., Shabbir M.Z., Kumar N., Malik Y.S., Singh R.K. (2015). Avian rotavirus enteritis —An updated review. Vet. Q..

[19] Otto P., Liebler-Tenorio E.M., Elschner M., Reetz J., Löhren U., Diller R. (2006). Detection of rotaviruses and intestinal lesions in broiler chicks from flocks with runting and stunting syndrome (RSS). Avian Dis..

[20] Otto P.H., Ahmed M.U., Hotzel H., Machnowska P., Reetz J., Roth B., Trojnar E., Johne R. (2012). Detection of avian rotaviruses of groups A, D, F and G in diseased chickens and turkeys from Europe and Bangladesh. Vet. Microbiol..

[21] Kindler E., Trojnar E., Heckel G., Otto P.H., Johne R. (2013). Analysis of rotavirus species diversity and evolution including the newly determined full-length genome sequences of rotavirus F and G. Infect. Genet. Evol..

[22] Phan T.G., Vo N.P., Boros Á., Pankovics P., Reuter G., Li O.T., Wang C., Deng X., Poon L.L., Delwart E. (2013). The viruses of wild pigeon droppings. PLoS ONE.

[23] Beserra L.A., Gregori F. (2014). Description of rotavirus F in broilers from Brazilian poultry farms. Avian Dis..

[24] Bodewes R., van Run P.R., Schürch A.C., Koopmans M.P., Osterhaus A.D., Baumgärtner W., Kuiken T., Smits S.L. (2015). Virus characterization and discovery in formalin-fixed paraffin-embedded tissues. J. Virol. Methods..

[25] Stucker K.M., Stockwell T.B., Nyaga M.M., Halpin R.A., Fedorova N., Akopov A., Ngoveni H., Peenze I., Seheri M.L., Mphahlele M.J. (2015). Complete genomic sequence for an avian group G rotavirus from South Africa. Genome Announc..

[26] Mascarenhas J.D., Bezerra D.A., Silva R.R., Silva M.J., Júnior E.C., Soares L.S. (2016). Detection of the VP6 gene of group F and G rotaviruses in broiler chicken fecal samples from the Amazon region of Brazil. Arch. Virol..

[27] Ramig R.F. (1997). Genetics of the rotaviruses. Annu. Rev. Microbiol..

[28] Jain S., Vashistt J., Changotra H. (2014). Rotaviruses: Is their surveillance needed?. Vaccine.

[29] Iturriza-Gómara M., Green J., Brown D.W., Ramsay M., Desselberger U., Gray J.J. (2000). Molecular epidemiology of human group A rotavirus infections in the United Kingdom between 1995 and 1998. J. Clin. Microbiol..

[30] Dóró R., Farkas S.L., Martella V., Bányai K. (2015). Zoonotic transmission of rotavirus: Surveillance and control. Expert. Rev. Anti. Infect. Ther..

[31] Matthijnssens J., Ciarlet M., Rahman M., Attoui H., Bányai K., Estes M.K., Gentsch J.R., Iturriza-Gómara M., Kirkwood C.D., Martella V. (2008). Recommendations for the classification of group A rotaviruses using all 11 genomic RNA segments. Arch. Virol..

[32] Matthijnssens J., Ciarlet M., Heiman E., Arijs I., Delbeke T., McDonald S.M., Palombo E.A., Iturriza-Gómara M., Maes P., Patton J.T. (2008). Full genome-based classification of rotaviruses reveals a common origin between human Wa-Like and porcine rotavirus strains and human DS-1-like and bovine rotavirus strains. J. Virol..

[33] Matthijnssens J., Ciarlet M., McDonald S.M., Attoui H., Bányai K., Brister J.R., Buesa J., Esona M.D., Estes M.K., Gentsch J.R. (2011). Uniformity of rotavirus strain nomenclature proposed by the Rotavirus Classification Working Group (RCWG). Arch. Virol..

[34] Kuga K., Miyazaki A., Suzuki T., Takagi M., Hattori N., Katsuda K., Mase M., Sugiyama M., Tsunemitsu H. (2009). Genetic diversity and classification of the outer capsid glycoprotein VP7 of porcine group B rotaviruses. Arch. Virol..

[35] Marthaler D., Rossow K., Gramer M., Collins J., Goyal S., Tsunemitsu H., Kuga K., Suzuki T., Ciarlet M., Matthijnssens J. (2012). Detection of substantial porcine group B rotavirus genetic diversity in the United States, resulting in a modified classification proposal for G genotypes. Virology.

[36] Suzuki T., Hasebe A. (2017). A provisional complete genome-based genotyping system for rotavirus species C from terrestrial mammals. J. Gen. Virol..

[37] Suzuki T., Inoue D. (2018). Full genome-based genotyping system for rotavirus H and detection of potential gene recombination in nonstructural protein 3 between porcine rotavirus H and rotavirus C. J. Gen. Virol..

[38] McNulty M.S., Todd D., Allan G.M., McFerran J.B., Greene J.A. (1984). Epidemiology of rotavirus infection in broiler chickens: Recognition of four serogroups. Arch. Virol..

[39] Kang S.Y., Nagaraja K.V., Newman J.A. (1986). Electropherotypic analysis of rotaviruses isolated from turkeys. Avian Dis..

[40] Theil K.W., Reynolds D.L., Saif Y.M. (1986). Genomic variation among avian rotavirus-like viruses detected by polyacrylamide gel electrophoresis. Avian Dis..

[41] Falcone E., Busi C., Lavazza A., Monini M., Bertoletti M., Canelli E., Vignolo E., Ruggeri F.M., Boniotti M.B. (2015). Molecular characterization of avian rotaviruses circulating in Italian poultry flocks. Avian Pathol..

[42] Duarte Júnior J.W.B., Chagas E.H.N., Serra A.C.S., Souto L.C.D.S., da Penha Júnior E.T., Bandeira R.D.S.E., Guimarães R.J.P.S., Oliveira H.G.D.S., Sousa T.K.S., Lopes C.T.A. (2021). Ocurrence of rotavirus and picobirnavirus in wild and exotic avian from amazon forest. PLoS Negl. Trop. Dis..

[43] Pinheiro M.S., Dias J.B.L., Cunha B.R.A.V., Petrucci M.P., Travassos C.E.P.F., Mendes G.S., Santos N. (2022). Rotavirus F and G circulating in chickens in Southeastern Brazil. Trop. Anim. Health Prod..

[44] Scorsato A.P., Telles J.E.Q. (2011). Factors that affect the quality of DNA extracted from biological samples stored in paraffin blocks. J. Bras. Patol. Med. Lab..

[45] Tamura K., Stecher G., Kumar S. (2021). MEGA11: Molecular Evolutionary Genetics Analysis Version 11. Mol. Biol. Evol..

[46] Gouvea V., Brantly M. (1995). Is rotavirus a population of reassortants?. Trends Microbiol..

[47] Martella V., Bányai K., Matthijnssens J., Buonavoglia C., Ciarlet M. (2010). Zoonotic aspects of rotaviruses. Vet Microbiol..

[48] Patton J.T. (2012). Rotavirus diversity and evolution in the post-vaccine world. Discov. Med..

[49] Degiuseppe J.I., Stupka J.A. (2020). Genotype distribution of Group A rotavirus in children before and after massive vaccination in Latin America and the Caribbean: Systematic review. Vaccine.

[50] Capua I., Alexander D. (2010). Perspectives on the global threat: The challenge of avian influenza viruses for the world’s veterinary community. Avian Dis..

[51] Trovão N.S., Shepherd F.K., Herzberg K., Jarvis M.C., Lam H.C., Rovira A., Culhane M.R., Nelson M.I., Marthaler D.G. (2019). Evolution of rotavirus C in humans and several domestic animal species. Zoonoses Public Health.

